# Inflammatory Networks in Renal Cell Carcinoma

**DOI:** 10.3390/cancers15082212

**Published:** 2023-04-09

**Authors:** Linus Kruk, Medina Mamtimin, Attila Braun, Hans-Joachim Anders, Joachim Andrassy, Thomas Gudermann, Elmina Mammadova-Bach

**Affiliations:** 1 Walther-Straub-Institute for Pharmacology and Toxicology, Ludwig-Maximilian-University, 80336 Munich, Germany; 2 Division of Nephrology, Department of Medicine IV, Hospital of the Ludwig-Maximilian-University, 80336 Munich, Germany; 3 Division of General, Visceral, Vascular and Transplant Surgery, Hospital of LMU, 81377 Munich, Germany; 4 German Center for Lung Research (DZL), 80336 Munich, Germany

**Keywords:** renal cell carcinoma, inflammation, immune cells, anti-inflammatory therapies

## Abstract

**Simple Summary:**

Inflammation is an important hallmark of advanced renal cell carcinoma and plays a pivotal role in tumor progression, metastasis, and anti-cancer resistance. Inflammation regulates the crosstalk between tumor cells and the surrounding tumor microenvironment, thereby promoting immune cell infiltration and release of inflammatory mediators and inducing immunosuppressive functions. Although inflammatory pathways sustain the function of renal cancer cells, the activatory effects are highly dependent on the immune cell landscape and the stage of RCC tumors. The aim of this review is to summarize the recent works describing the role of inflammation in the growth and metastasis of RCC tumors. This review also critically discusses the clinically relevant inflammatory pathways as potential targets for disease prevention and treatment.

**Abstract:**

Cancer-associated inflammation has been established as a hallmark feature of almost all solid cancers. Tumor-extrinsic and intrinsic signaling pathways regulate the process of cancer-associated inflammation. Tumor-extrinsic inflammation is triggered by many factors, including infection, obesity, autoimmune disorders, and exposure to toxic and radioactive substances. Intrinsic inflammation can be induced by genomic mutation, genome instability and epigenetic remodeling in cancer cells that promote immunosuppressive traits, inducing the recruitment and activation of inflammatory immune cells. In RCC, many cancer cell-intrinsic alterations are assembled, upregulating inflammatory pathways, which enhance chemokine release and neoantigen expression. Furthermore, immune cells activate the endothelium and induce metabolic shifts, thereby amplifying both the paracrine and autocrine inflammatory loops to promote RCC tumor growth and progression. Together with tumor-extrinsic inflammatory factors, tumor-intrinsic signaling pathways trigger a Janus-faced tumor microenvironment, thereby simultaneously promoting or inhibiting tumor growth. For therapeutic success, it is important to understand the pathomechanisms of cancer-associated inflammation, which promote cancer progression. In this review, we describe the molecular mechanisms of cancer-associated inflammation that influence cancer and immune cell functions, thereby increasing tumor malignancy and anti-cancer resistance. We also discuss the potential of anti-inflammatory treatments, which may provide clinical benefits in RCCs and possible avenues for therapy and future research.

## 1. Introduction

Renal cell carcinoma (RCC) accounts for 95% of kidney-related malignancies and is considered a heterogeneous group of diseases that are classified according to their histological characteristics [1,2]. The clear-cell RCC (ccRCC) is the most predominant form, followed by non-clear-cell RCCs such as papillary (pRCC) and chromophobe (chRCC), clear-cell papillary (ccpRCC), collecting duct (cdRCC) and medullary (mRCC) and unclassified (uRCC) RCC types [2]. Although the ontogenesis of RCC is not fully elucidated, RCC can be either sporadic (non-inherited) or familial (inherited) and associated with distinct genetic mutations. The most frequently implicated genes in the pathogenesis of ccRCC are von Hippel–Lindau (*VHL*), *PBRM1*, *SETD2*, *BAP1*, *KDM5C* and *MTOR*. Non-clear-cell RCCs present similar genetic alterations with additional genetic mutations such as *CDKN2A, NRF2, PTEN, TP53, TFEB, TFE3* and *SMARCB1* and with numerous mitochondrial gene dysfunctions [1]. RCC possesses the highest number of indel mutations on a pan-cancer basis. Sporadic RCC can arise as a consequence of kidney injury where injury-related deoxyribonucleic acid (DNA) damage introduces somatic mutations, and subsequent proliferative healing responses drive the monoclonal expansion of mutated epithelial cell clones, e.g., in pRCC. These genomic changes of RCC induce the release of high numbers of tumor-specific antigens (neo-antigens), thereby eliciting the adaptive immune response [3,4,5]. RCC is a highly vascularized tumor, susceptible to the recruitment of immune and stromal cells, forming an inflammatory microenvironment [6,7]. The early stage of RCC is treated by curative surgical resection. The treatment of advanced and metastatic RCC is difficult, based on the combination of several agents, targeting multiple receptor tyrosine kinases, including growth-factor receptors such as vascular endothelial growth factor receptor (VEGFR), or directly targeting VEGF, often in combination with immune checkpoint inhibitors (ICIs) [8,9,10,11,12,13]. The heterogeneity of the genetic background and tumor microenvironment (TME) varies the treatment response among patients [14]. Cancer-associated inflammation is the most recurrent hallmark of many solid cancer types. Recent studies have linked inflammation to metastatic progression in patients with RCCs [15,16,17,18,19,20]. Cancer cell-extrinsic pathological conditions, such as systemic and local infections and inflammatory diseases, can activate oncogenic pathways leading to the development of solid tumors. 

On the other hand, cancer cell-intrinsic oncogenic or epigenetic changes lead to pro-inflammatory reactions, including the release of cytokines and growth factors into the TME, thereby inducing cancer progression [21]. Accordingly, high levels of pro-inflammatory cytokines and consequent enhanced systemic inflammatory responses are observed. Elevated C-reactive protein concentration and hypo-albuminuria, elevated neutrophil and platelet counts, and a high neutrophil-lymphocyte ratio have been shown to correlate with poor RCC survival [15,16,17,18,19,20,22,23,24]. In TME, intricate crosstalk between cancer and host cells (i.e., stroma, endothelial, immune cells) induces an immune response, thereby modulating tumor growth [25]. ccRCC tumors are characterized by rich leukocyte infiltrates and an abundance of intratumoral CD8^+^ and CD4^+^ T cells, which are associated with high tumor grade and shorter patient survival [26]. RCC tumors express human leukocyte antigen G (HLA-G), and typically, the loss of major histocompatibility complex I (MHCI), which correlates with inhibition of CD8+ T cells, induction of regulatory T cells (T_reg_) and inhibition of natural killer cells (NKs) and dendritic cells (DCs) [27,28,29]. Studies analyzing the TME-associated gene expression signature could divide RCC into non-inflamed and inflamed subtypes. Based on the histological analysis of RCC, the non-inflamed subtype is characterized by increased angiogenesis and plasmacytoid DCs and mast cell infiltrates, while the inflamed subtype showed high levels of complement system proteins and infiltrates of different immune cell types, such as T_reg_, NKs, Th, CD8^+^, B and activated DCs, as well as neutrophils, macrophages and eosinophils [30,31,32]. The inflamed subtype is frequently associated with the clinical manifestation of thrombocytosis or anemia, which account for worse patient survival, highlighting the impact of renal and systemic inflammation on the progression of RCCs and patient outcome [30,31,32]. In this review, we describe the molecular mechanisms of inflammation that influence tumor cell function and immune cell activity, thereby increasing tumor malignancy and anti-cancer resistance. We also discuss potential anti-inflammatory treatments of RCCs, which may provide clinical benefits in patients with advanced tumor and metastases. 

## 2. Inflammatory Signaling Pathways in RCC

**Figure 1:**
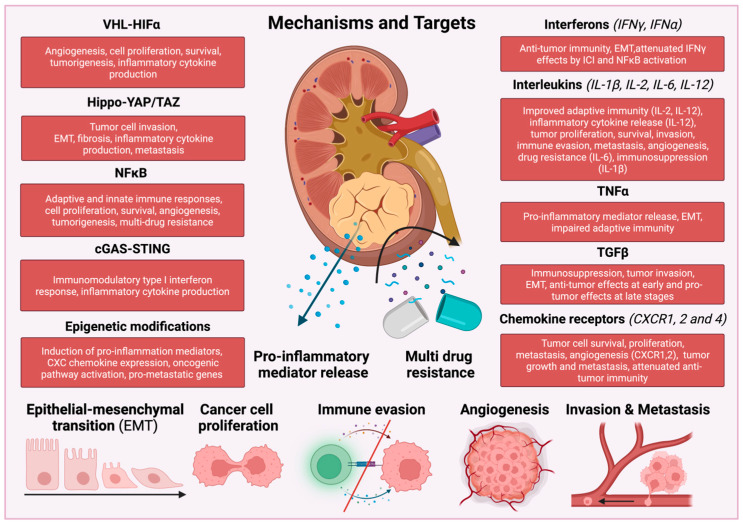
Schematic overview of molecular mechanisms and potential targets of RCC-associated inflammation. Intricate crosstalk between renal cancer cells and TME, regulates pro-inflammatory mediator release, angiogenesis, tumor cell plasticity, survival and proliferation, immune evasion, thereby leading to drug resistance, tumor progression and metastasis.

RCCs produce and release several pro-inflammatory cytokines that activate different subtypes of immune cells, thereby promoting tumor progression and metastasis. In RCC tumors, activation of transcription factors, such as nuclear factor kappa-light-chain-enhancer of activated B cells (NF-κB), signal transducer and activator of transcription 3 (STAT3) and hypoxia-inducible factor 1α (HIF1α) increase the production of angiogenic factors and inflammatory mediators, such as interleukins IL-1β, IL-6, IL-23 and tumor necrosis factor α (TNFα) and chemokines, creating a cancer-associated inflammatory TME [33,34,35] (Figure 1).

Tumor cell-extrinsic inflammation can be triggered by many factors, including infection, obesity, autoimmune disorders, and exposure to tobacco and toxic substances [36]. Inflammation can also be induced by epigenetic remodeling of paracrine or autocrine signaling, genomic mutation, genome instability of cancer cells, called tumor-intrinsic inflammation, that fuel immunosuppressive or tumor-promoting traits, leading to the recruitment and activation of inflammatory cells [36]. Although due to the complexity of the molecular mechanisms, the distinction between systemic response and tumor-intrinsic activity is challenging.

## 3. vHL-HIFα

The majority of sporadic ccRCCs represent mutations in the von Hippel–Lindau tumor-suppressor gene (*VHL*). Therefore, *VHL* was considered a main target in conventional therapy of ccRCC [37]. The protein VHL is responsible for targeting the alpha (α) subunits of hypoxia-inducible transcription factors (HIF1α and HIF2α) for degradation through a process that depends on oxygen and ubiquitin [38,39]. VHL protein can destabilize hypoxia-induced genes, thereby influencing cellular and tissue response to hypoxia, such as VEGF-mediated angiogenesis, energy metabolism through glucose transporter 1 (GLUT1), or production of platelet-derived growth factor subunit B (PDGFB) [40]. Additionally, VHL protein activates signaling pathways that control the production of several inflammatory cytokines, including TNFα, chemokine receptor CXCR4, and NF-κB [41,42,43].

When VHL is genetically inactivated, HIF1α and HIF2α become stabilized, which leads to changes in cellular metabolism, angiogenesis, epithelial-mesenchymal transition (EMT), invasion, and tumor metastasis that contribute to the progression of ccRCC [44]. HIF2α is believed to have a pro-tumorigenic role in ccRCC, while HIF1α inhibits aggressive tumor behavior, thereby acting as a tumor suppressor. The loss of the region of chromosome 14q that harbors the *HIF1A* gene is associated with poor survival and is often found in ccRCC metastases [45]. ccRCC tumors expressing only HIF2α have higher proliferation index than those expressing both HIF1α and HIF2α [46]. ccRCC tumor cell lines frequently exhibit intragenic deletions of *HIF1A* but express wild-type HIF2α [47]. HIF2α is necessary for the growth of ccRCC xenografts, whereas knockdown of HIF1α enhances xenograft tumor formation in cell lines expressing both HIF1α and HIF2α [39,47,48]. Therefore, various therapeutic approaches have been elaborated to target HIF2α signaling routes in ccRCC [49,50]. A recent study based on the introduction of floxed alleles of HIF1α and HIF2α into an autochthonous mouse model of ccRCC challenged this concept [51]. It was shown that the presence of HIF1α is essential for the formation of tumors, whereas the absence of HIF2α has a limited effect on tumor initiation and growth [51]. Although HIF1α plays a crucial role in glycolysis, HIF2α regulates the expression of genes that are involved in various cellular processes, such as lipoprotein metabolism, ribosome biogenesis, and transcriptional activities of E2F and MYC [51]. Interestingly, in this study, HIF2α-deficient tumors were also characterized by increased antigen presentation, interferon (IFN) signaling, and infiltration and activation of CD8+ T cells [51]. 

Abolished *VHL* gene expression in mice induces hyperplastic clear cell lesions with severe inflammation and fibrosis [52]. This inflammatory response was associated with increased inositol-requiring transmembrane kinase/endoribonuclease 1α (IRE1α) kinase activity required for the polarization and recruitment of inflammatory macrophages [53]. IRE1α signaling pathway in macrophages drives obesity and metabolic syndrome through impairing brown adipose tissue activity and white adipose tissue browning [54]. *VHL* deficiency induces the infiltration of macrophages and their polarization toward M2 pro-inflammatory phenotype. Consequently, these macrophages enhance tumor progression and metastasis by secreting high amounts of chemokine (C-C motif) ligand 18 (CCL18) and transforming growth factor beta 1 (TGFβ1) [55]. 

AXL is a receptor tyrosine kinase expressed in different cancer cell types, and the gene expression is regulated by HIF1α and HIF2α in hypoxic cancer cells lacking VHL protein [56]. Therefore, invasion and metastasis of ccRCC are tightly regulated by AXL signaling route. Downregulation of *AXL* gene expression or competitive inhibition of AXL function with a soluble variant of AXL (sAXL) has an inhibitory effect on the metastasis of ccRCC cells [57]. Several tyrosine-kinase inhibitors (TKIs) or monoclonal antibodies (bevacizumab, sunitinib and sorafenib) that block VEGFA mediated tyrosine-kinase pathways have been used to treat advanced RCC [10,58,59]. However, only a few patients positively responded to this treatment with long-lasting tumor suppression because the majority of RCC tumors use an alternative “angiogenic escape” pathway, thereby sustaining tumor revascularization [60]. AXL and fibroblast growth factor receptor (FGFR) are involved in this alternative pathway since increased levels of FGFR and AXL are directly associated with cancer cell resistance to the TKI (sunitinib) [61]. Therefore, the other TKI, lenvatinib, was developed, which effectively inhibited different isoforms of FGFR and vascular endothelial growth factor receptor (VEGFR) [61,62]. The combination of lenvantinib with pembrolizumab or everolimus have been successfully used in the treatment of advanced RCC [63,64]. Another therapeutic strategy was developed to target HIF pathways [65]. Belzutifan is a small-molecule inhibitor of HIF2α, which disrupts the heterodimerization of HIF2α with HIF1β, required to form an active transcription factor [66]. Several clinical studies evaluated the effects of belzutifan in RCCs [66], showing improved bioavailability and efficacy compared to other HIF inhibitors [67]. Recently, belzutifan was approved by the Food and Drug Administration (FDA) for adult patients with VHL-associated RCC, central nervous system hemangioblastomas, or pancreatic neuroendocrine tumors without the need for immediate surgery [68]. 

Interestingly, HIF2α is recruited by the PAX-8-bound transcriptional enhancers such as cyclin D1 (*CCND1*) and *MYC* [69,70]. In ccRCC, rs7948643 is a common genetic variant linked to the significant renal cancer risk locus rs7105934 on chromosome 11q13.3, which coincidently falls within PAX8-binding site E11:69419. Both PAX8 and HIF2α play an important role in E11:69419 transcriptional activity, and genetic inactivation of *PAX8* is tolerated in the mouse kidney leading to the assumption that PAX8 synergistically to HIF2α could be a therapeutic target in ccRCC [69]. The synthetic lethality of VHL combined with many additional genetic alterations, offers another therapeutic avenue [71]. For instance, disruption of *VHL* and *EZH1*, *SLC2A1*, *ROCK1*, *SKP2*, *AURKA*, or *TBK1* genes can each inhibit cancer progression by inducing the death of tumor cells [72,73,74,75,76,77,78]. A recent study discovered that HIF2α accumulation could enhance the gene expression of proliferative CDK4/6 partner cyclin D1 while the synthetic lethality of VHL-/- and cyclin-dependent kinases 4 and 6 (CDK4/6) is independent of HIF2α accumulation [77]. This may therapeutically represent benefits since HIF2α-inhibitors are only effective in a small group of ccRCC patients, and a large cohort of ccRCC patients have pre-existing or acquired resistance to HIF2α-inhibitors [49,77]. Therefore, future studies are needed to determine whether inhibition of CDK4/6 is a suitable therapeutic approach for ccRCC, alone or in combination with HIF2α inhibitors [77].

## 4. Hippo-YAP/TAZ

Several studies have linked RCC and inflammation with the Hippo pathway. The core components of the Hippo pathway consist of the MST1/2 and LATS1/2 kinases [79]. Downstream from the Hippo pathway are the YAP/TAZ transcription coactivators, which are suppressed through phosphorylation by MST1/2 and LATS1/2 kinases [79]. The Hippo pathway is regulated by the merlin/neurofibromatosis type 2 (NF2), a well-studied tumor suppressor gene. NF2 enhances the Hippo signaling pathway by inhibiting YAP/TAZ nuclear translocation through phosphorylation, isolation, and degradation [79]. 

Changes in the biomechanical properties of cells and tissues can activate the YAP/TAZ sensor, leading to the release of pro-inflammatory and pro-fibrotic signals. YAP plays a critical role in fibrosis following ischemic acute kidney injury (AKI) [80] and numerous studies have linked aberrant YAP activation to kidney fibrosis [81,82,83,84]. Wnt5a has been shown to stimulate renal fibrosis by inducing M2 macrophage polarization through YAP/TAZ activation [85]. The polyploidization of kidney tubular cells is considered a rapid mechanism to increase the residual function of differentiated tubular cells during the early phase of injury, thereby avoiding AKI fatality. However, during the late phase of AKI, the cycling polyploid kidney tubular cells were shown to become senescent, developing a pro-fibrotic phenotype that promotes chronic kidney disease (CKD) [86]. Interestingly, the blockade of YAP1 and TEAD coactivator transcriptional activity using CA3 (CIL56) during the recovery phase of AKI was proposed as a promising therapeutic approach to prevent CKD [86].

Abnormal Hippo pathway signaling and YAP activation are observed in NF2-deficient tumors, including uRCC [87,88]. Silencing YAP/TAZ in NF2-deficient tumors induces tumor regression by increasing mitochondrial respiration, reducing glycolic-dependent growth, and causing the accumulation of reactive oxygen species (ROS) and consequent oxidative stress-induced cell death [89]. LATS1 kinase has been proposed as a tumor suppressor and is reduced in many types of cancer, including RCC [90,91]. Consistently, high expression of LATS1 or LATS2 is associated with longer disease-free survival (DFS) and overall survival (OS) in RCC patients [92]. Patients with ccRCC also exhibit a significant increase in TAZ expression, which is associated with poor prognosis [93]. It was also shown that TAZ deletion can counteract ferroptosis, which regulates cell death [94]. TAZ regulates the expression of epithelial membrane protein 1 (EMP1), which induces the expression of nicotinamide adenine dinucleotide phosphate (NADPH) oxidase 4 (Nox4)—an enzyme essential for ROS-mediated ferroptosis in the kidneys [94]. Hypoxia-induced Nox4 and NADPH were shown to increase IL-6 and IL-8 levels in RCC, thereby increasing the invasion of cancer cells, [23] indicating a positive feedback loop between IL-6/8 and YAP/TAZ during the progression of RCC. 

The Hippo pathway can also interact with the epidermal growth factor receptor (EGFR) pathway, thereby regulating cancer cell invasion [95]. EGFR was shown to downregulate claudin-2, a type of tight junction protein. Its downregulation results in the disruption of claudin-2-YAP interaction, thereby leading to the activation of YAP and oncogenic proteins that induce mesenchymal plasticity of cancer cells, thereby promoting tumor metastasis [95]. In sporadic ccRCC, the expression of tight junction proteins occludin, claudin 1 and E-cadherin is downregulated upon the loss of VHL [96]. Although the overexpression of occludin and claudin 1 could restore the assembly of tight junctions, the re-expression of E-cadherin was insufficient to restore this process [96]. Altogether, these studies indicated the role of the activated YAP pathway in the disruption of epithelial polarity and consequent metastasis.

High-grade ccRCC is associated with the downregulation of salvador homolog 1 (SAV1), which is a tumor suppressor and a component of the Hippo signaling pathway [97,98]. In contrast, the overexpression of YAP1 was associated with the pathogenesis of mucinous tubular spindle cell carcinoma (MTSCC), a rare subtype of renal cell carcinoma [99]. The use of YAP inhibitors, such as verteporfin (VP), was proposed as a treatment for patients with rare MTSCC with sarcomatoid differentiation or metastatic disease [88,99].

The leukemia inhibitory factor receptor (LIFR) is a potential downstream target of the Hippo pathway. Knockdown of the LIFR gene was shown to increase YAP expression and inhibit Hippo signaling pathway kinase activity, thereby promoting tumor metastasis [100]. SH3 domain-binding glutamate rich protein-like 2 (SH3BGRL2) is involved in the suppression of EMT and metastasis in ccRCC tumors by inhibiting the nuclear translocation of YAP and its interaction with the TEAD coactivator in the nuclei [101]. Conversely, the knockdown of SH3BGRL2 activates the Hippo pathway by binding TEAD, leading to the downregulation of Twist1 and the shifting EMT, ultimately inhibiting the growth and metastasis of RCC cells [101]. Recently, CA3 (CIL56), YAP1/TEAD inhibition was shown to strongly inhibit esophageal adenocarcinoma cell growth and induce strong anti-tumor effects by suppressing tumor cell proliferation, and cancer stem cell phenotype in a xenograft model with no apparent toxicity [102]. However, CA3 (CIL56) has not yet been tested in experimental models of RCC. A Phase 1 study has been planned to study the effect of IK-930, an oral TEAD inhibitor targeting the Hippo pathway in patients with advanced tumors, including RCC (NCT05228015).

## 5. NF-κB Pathway

The nuclear factor kappa-light-chain-enhancer of activated B cells (NF-κB) is a transcription factor ubiquitously expressed in almost all mammalian cells, forming heterodimers [103]. The classic “canonical” NF-κB pathway forms a complex with p50 and RelA, while the non-canonical NF-κB pathway is predominantly associated with p52/RelB [103]. NF-κB pathways are involved in response to inflammatory stress, which are activated by cytokines, bacterial toxins, viral products and cell death stimuli. Although NF-κB pathways regulate the innate immune system in healthy conditions, abnormal NF-κB activation can trigger abnormal inflammatory responses during tumor development [104]. NF-κB pathways enhance cancer cell migration and invasion by upregulating multi-drug resistance genes, pro-angiogenic factors and pro-inflammatory cytokines such as VEGFA, EGF, IL-6 and IL-8 [105]. Interestingly, the NF-κB subunit p50 regulates macrophage function in cancer, and its inhibition converts the pro-tumor phenotype to M1-like cytotoxic phenotype and consequently attenuates tumor growth [106]. VHL loss could activate NF-κB pathways [107], thereby inducing gene expression of anti-apoptotic Bcl-xL and Bcl-2, and inhibiting tumor suppressor p53, highlighting the potential role in RCC development [108]. Studies showed that elevated NF-κB expression correlated with a low rate of patient survival with RCC [109]. Activation of NF-κB and STAT3 pathways enhances tumor infiltration of M1/M2-like macrophages and T_reg_ cells, thereby increasing tumor growth and metastasis in RCC [110].

N6-adenosine-methyltransferase (METTL3) is the catalytic subunit of the m6A methyltransferase complex, which is involved in the post-transcriptional methylation of adenosine residues of mRNAs. In tumor-infiltrating myeloid cells, METTL3 regulates the induction of NF-κB through the ERK pathway and response to programmed cell death protein 1 (PD-1) checkpoint blockade [111]. Therefore, it was proposed that a blockade of NF-κB activation in myeloid-derived cells and T_reg_ cells may offer a therapeutic avenue by inducing an immunosuppressive response. Contrary, a recent study showed that adoptive cell transfer of T cells with constitutively active upstream NF-κB activator IκB kinase β (IKKβ) increased the number of functional CD8(+) T cells that are selective for tumors and enhanced cytotoxic activity [112]. This demonstrated the bivalent role of NF-κB targeting when applied to specific immune cell subsets.

Bortezomib is a proteasome inhibitor with anti-tumor activity blocking NF-kB function. Bortezomib increases apoptosis, decreases angiogenic cytokine production, and inhibits cancer cell adhesion to stroma [113]. Although bortezomib was used in the treatment of patients with advanced RCCs [114,115], similar to other NF-κB inhibitors, it also has off-target effects. [113,116,117]. Bortezomib enhances caspase-8 activity, sensing RCC to TNF–related apoptosis-inducing ligand (TRAIL). Without priming caspase-8 activation, bortezomib has limited efficiency to induce TRAIL-mediated cancer cell apoptosis [115].

## 6. cGAS-STING and BAF Complex

The cGAS–STING signalosome is composed of cyclic GMP-AMP synthase (cGAS) and the stimulator of IFN genes (STING), which detect cytoplasmic DNA in virus-infected cells and triggers IFN regulatory transcription factor 3 (IRF3)-dependent inflammatory gene expressions, thereby accelerating the innate immune response [118]. Activated STING is translocated to the Golgi and activates TANK-binding kinase 1 (TBK1), which further phosphorylates and activates IRF3. STING can also activate MAPK and NF-κB pathways, thereby increasing the production of inflammatory cytokines and chemokines [118]. Although cGAS–STING signalosome is important in the DNA detection of pathogens, it also binds endogenous DNA, which is released from the nucleus of cancer or apoptotic cells. Mitochondria dysfunction and mtDNA release also activate the cGAS-STING pathway. Therefore, the cGAS-STING signalosome was proposed to be contributed to anti-tumor immunity by inducing an immune suppressive TME [119]. Sensing of cytosolic DNA in DCs can sustain anti-tumor activity by inducing IFN production, priming cytotoxic CD8+ T cells and triggering cancer cell senescence. Interestingly, this senescence-associated secretory phenotype eliciting the release of pro-inflammatory cytokines, chemokines, proteases, and growth factors, is shown to restrict tumorigenesis [120]. *ENPP1*, a transmembrane phosphodiesterase in purinergic signaling, can downregulate cGAS-STING signaling by hydrolyzing cyclic guanosine monophosphate–adenosine monophosphate (cGAMP) [121]. Interestingly, brain metastatic cancer cells can activate cGAS-STING signalosome, transferring cGAMP to neighboring astrocytes, thereby increasing the production of inflammatory cytokines such as IFN and TNF and consequently promoting metastasis [120]. 

Recently, cGAS-STING was also described as a risk factor in different types of RCCs. The score of differentially expressed genes associated with cGAS-STING was shown to be correlated to poor OS and was significantly higher compared to other cancer types [122]. This result suggests a unique TME of RCCs, which is differently regulated by cGAS-STING signalosome, thus promoting tumor formation and metastasis. The effect of cGAS-STING activation appears to be highly dependent on the disease context. In AKI and CKD, STING induction in mice leads to albuminuria and podocyte loss [123]. Supporting this ambivalent role of cGAS-STING, the novel STING antagonist, H151, significantly ameliorates renal function, kidney morphology, and renal inflammation in cisplatin-induced AKI [124].

Currently, several clinical trials are undergoing to explore the efficacy of STING agonists and DNA damage repair (DDR) targeting drugs in patients with different kidney cancer, including mRCC and metastatic ccRCC (NCT03274258; NCT03587662; NCT03010176). The impact of cGAS-STING targeting drugs in the cancer therapy of RCC remains to be addressed by future studies. Especially, contributing pathways with over-activation, and off-target effects of agents on immune cells have not yet been fully understood [125]. 

The histone-packed nucleosomes need to be opened to expose chromatin-free gene regulatory elements for transcription factors to maintain gene expression [126]. Nucleosome repositioning is regulated by the chromatin-remodeling complex BAF [126]. Interestingly, many cancer cells carry BAF mutations indicating an important tumor-suppressive role in humans [126]. The SWI/SNF related, matrix-associated, actin-dependent regulator of chromatin, subfamily B, member 1 (SMARCB1) protein is a subunit of the BAF complex. SMARCB1 deficiency resulted in the miss-localization of the BAF complex, which leads to the inhibition of gene expression [127]. However, residual BAF complex can still maintain abnormal gene expression required for cancer progression. SMARCB1 deficiency has been detected in mRCC [128]. SMARCB1 deficiency leads to c-MYC pathway activation and increased DNA replication stress. Consequently, a highly inflammatory phenotype of mRCC with elevated replication stress was associated with the activation of the cGAS-STING innate immune pathway [128]. The potential therapeutic value of targeting the DDR enzyme, poly(ADP-ribose) polymerase (PARP) pathway, alone or in combination with platinum chemotherapy, has been suggested in mRCC [128]. 

The SWI/SNF protein complex mobilizes nucleosomes that are required for kinetochore localization and also modifies chromatin structure through the hydrolysis of ATP molecules [126]. *PBRM1* gene encodes a bromodomain-containing protein (BAF180), which is a member of the DNA-targeting subunit of the SWI/SNF complex, recognizing acetylated histone residues, thereby localizing the complex in a specific chromatin region [129]. In addition to *VHL*, *PBRM1* is the most common mutated gene in ccRCC. PBRM1 deficiency results in increased proliferation, migration, and colony formation of ccRCC [130]. Although the exact pathophysiological role of PBRM1 in ccRCC has not been established, PBRM1 deficiency disturbs p53-dependent chromatin regulation; therefore, ccRCC tumors are able to escape from p53-mediated surveillance [131]. Inhibition of PARP or ATR improved synthetically lethal in PRBM1-deficient cells and induced, rather than attenuate the cGAS-STING pathway to elicit immunomodulatory type I IFN response [132]. 

Adaptive immunotherapy using chimeric antigen receptor T-cells (CAR-T), genetically engineered T cells expressing antigen-specific non-major histocompatibility complex-restricted receptors is a popular approach in immunotherapy of cancers, including RCCs [133]. CD70 is a member of a type II transmembrane cell-surface protein of the TNF receptor family, which is highly expressed in RCCs and metastatic tissues [134]. Many patients with ccRCC showed abnormally high CD70 expression [135]. Therefore, targeting of CD70 function using CAR-T therapy thus far has only modest benefits, and positively evaluated [136,137]. 

## 7. Epigenetic Mechanisms in RCC

Epigenetic mechanisms strongly influence gene expression in mammalians. Several histone-modifying enzymes such as histone acetyltransferases (HATs) and histone deacetylases (HDACs) can regulate promoter activity and chromatin condensation processes by the addition or removal of methyl- and acetyl groups [138]. Gene expression profiles of cancer cells can be drastically modified by epigenetic mechanisms, which propagate tumor progression [139]. Mediators of systemic inflammation, such as nitric oxide, prostaglandins, or cytokines (IL-1β and IL-6), also modify gene expression profile through the activation of DNA methyltransferases (DNMTs) and HDACs, thereby influencing the behavior of cancer cells [140]. The inflamed TME can further change the epigenome in immune and epithelial cells, thereby influencing innate and adaptive immune responses [141].

Epigenetic mechanisms regulate different steps of RCC development since more than 200 genes have been identified with abnormal gene methylation [142]. RCC-related genes such as *PBRM1*, *BAP1* and *SETD2* are tightly regulated by histone modification and nucleosome and chromatin remodeling [143]. Recently, a predictive classifier was presented, accurately stratifying RCC patients’ risk based on methylation of five CpG-regions independent of standard clinical prognostic factors [144]. These studies revealed the activation of NF-κB and CCAAT/enhancer-binding protein (C/EBP) pathways, inducing the demethylation of inflammation-related genes in advanced ccRCC [145,146]. HIF, NF-κB and C/EBP pathways can strongly upregulate gene expression of CXC chemokines during cancer progression, thereby resulting in cancer-cell-intrinsic inflammation [147,148]. This inflammatory signal induces epigenetic changes in ccRCC cells, resulting in a “super-enhancer” formation in the genome at chromosome 4, forming a growth-regulated oncogene region [147]. HIF2α and NF-κB pathways also play an important role in RCC metastasis, due to the activation of metastasis-associated enhancer (MAE), which amplifies the expression of chemokine receptor CXCR4, thereby inducing pro-metastatic activity [148].

Lysine-specific histone demethylase 1 (LSD1) is a flavin-dependent monoamine oxidase, which targets mono- and di-methylated lysines on histone 3 (H3K4 and H3K9). LSD1 was detected in half of the patients with ccRCCs. Histone methylation status changed in LSD1-knockdown kidney cancer cells, suggesting an epigenetic control, probably through androgen receptor transcription factors [149]. High LSD1 expression suppresses H3K4 methylation in RCCs, which dysregulates the anti-proliferative check-point regulator p21 [150]. Consequently, inhibition of LSD1 function could restore p21 levels, thereby inducing G1/S cell-cycle arrest in RCCs [150]. Therefore, LSD1 inhibitors were developed such as raloxifene [151] or tranylcypromine derivates [152] and other preclinical tested compounds (ORY-1001 [153], SP2095 [150]). Although no clinical trials with LSD1 inhibitors have been published in patients with RCCs, some compounds are clinically validated in other solid cancer types (NCT02959437, NCT05268666, NCT02712905).

VHL deficiency can stabilize HIF2α function at tumor-specific gain enhancers thereby upregulating the expression of ccRCC-related oncogenes, such as ZNF395 [154]. Class II HDAC inhibitors were proposed to induce anti-tumor effects through the degradation of HIF isoforms [155]. Therefore, early clinical trials used panobinostat as a non-selective pan-HDAC inhibitor, a drug that suppresses the proliferation of immature epithelial progenitor cells in the adult kidney and enforces their differentiation. Later, monotherapy with HDAC inhibitor vorinostat, or combined therapy with mTOR inhibitor were more effective in patients with RCCs than panobinostat alone (NCT00278395, NCT00324740, NCT00324870). Hence, the numbers of clinical studies are limited, future investigations are needed to determine the effectiveness of epigenetic modulators in RCCs, which may improve patient survival.

## 8. Inflammatory Immune Cell Landscape of RCC

**Figure 2:**
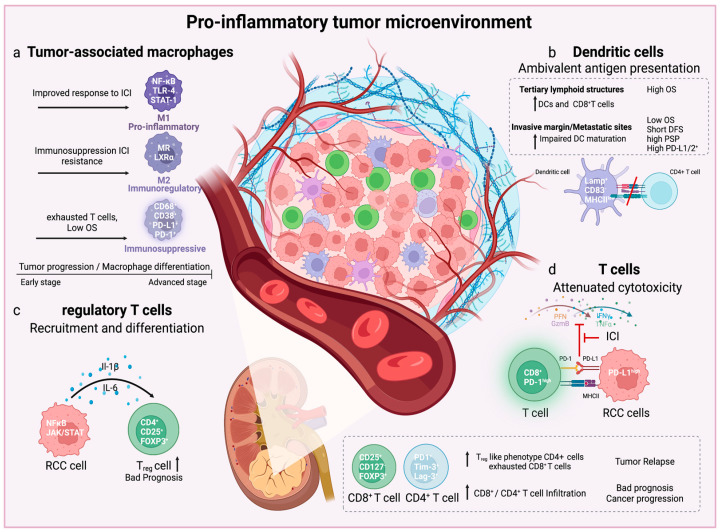
(**a**) Macrophages express a variety of markers, including pro-inflammatory, immune-regulatory, and suppressive markers. While M1 macrophages are responsible for the eradication of pathogens and tumor cells, M2 macrophages have immune suppressive properties inducing an exhausted signature in CD8+ T cells as well as the tendency for tissue repair and tumor progression. (**b**) DCs with impaired maturation (DC^Lamp^; DC^CD83-^; DC^MHCII,Low^) are located outside of tumor-associated tertiary lymphoid structures (NTLS) of RCC tumors and are associated with high levels of PD-L1/PD-L2 and poor prognosis. (**c**) T reg cells reduce the cytotoxicity of effector T cells, thereby inducing immunosuppressive phenotype in RCC tumors. (**d**) RCC tumors are abundant in immune cell infiltrates. In contrast to other solid tumor types, infiltration of CD8 + and CD4 + T cells into the TME is often associated with a poor prognosis and advanced tumor grade. Furthermore, inflammatory TME contains an elevated number of polyclonal exhausted CD8^+^Tim-3^+^/Lag-3^+^ TIL, associated with strong infiltration of dysfunctional DCs. PSP: pseudoprogression.

The immune cell landscape is regulated by tumor cell-presenting antigens, immune cell infiltration and immunomodulatory molecules in the TME, which are all engaged in a complex interplay between cancer cell internal and external factors, that influence tumor progression [156], (Figure 2). The immune cell landscape is an important factor contributing to the efficacy of cancer treatments, such as TKIs or ICIs, that target the TME of RCCs [25,157]. Numerous studies described ccRCC as an invasive cancer type with highly enriched T cell infiltrates. An immune profiling study of human ccRCC samples, identified T cells as the main immune cell population, followed by myeloid cells, NKs and B cells. However, in contrast to many other solid tumor types, infiltration of CD8^+^ and CD4^+^ T cells in ccRCC tumors is often linked to a poor prognosis and advanced tumor grade [25,158,159,160,161,162,163,164,165,166,167], highlighting T cell dysfunction during the progression of RCC. Interestingly, the presence of lymphocytes positive for PD-1 and tumor cells positive for programmed cell death-ligand 1 (PD-L1) and/or PD-L2 in the invasive margin of ccRCC was correlated with the shortest DFS, OS and higher risk of post-surgical cancer progression [168].

Dysfunctional maturation of DCs is characterized by the expression of lysosomal-associated membrane protein LAMP (DC^Lamp+^), CD83 (DC^CD83-^) and MHC class II protein (DC^MHCII,Low^) Such DCs are located outside of tumor-associated tertiary lymphoid structures (NTLS-DC), particularly in the invasive margins of primary ccRCC tumors [168]. Increased density of NTLS-DC at the invasive margins was associated with higher levels of PD-L1+ and/or PD-L2+ and a bad prognosis, whereas high levels of CD8+ cells and low levels of DCs in tertiary lymphoid structures (TLS-DC) served as the strongest independent variable for a poor outcome. A higher density of TLS-DC in CD8^+high^ groups was associated with a good prognosis [168]. Of note, patients’ outcomes were not significantly affected by the immunological profile of DCs in the tumor center. Tumor cells instigate a pro-inflammatory microenvironment, which leads to the recruitment and polarization of T cells towards an exhausted phenotype, potentially due to the presence of dysfunctional DCs in the TME outside of their canonical priming sites [168]. Shorter OS correlated to high densities of CD8^+^ T cells and DC^Lamp+^ cells at metastatic sites, while higher densities of NKs were linked to improved survival [162]. Additionally, intratumoral PD-1 expression was correlated with tumor size, nuclear grade, TNM stage, coagulative tumor necrosis, a sarcomatoid phenotype and a higher risk of cancer-related death [169]. 

Several transcriptomic studies have been conducted to classify metastatic ccRCCs, and results identified four subtypes (*ccrcc1* to *4). Crcc2* and *3* groups responded better to sunitinib therapy in comparison to *ccrcc1* and *4* groups. The latter two included less differentiated cancers with upregulated MYC targets and displayed a hyper-methylated gene profile associated with polycomb stem-cell-like phenotype. The *ccrcc4* group exhibited a sarcomatoid, pro-inflammatory, Th1-oriented phenotype with elevated B, T, and cytotoxic-cell-specific transcripts. The *ccrcc4* group also displayed an immune suppressive TME with high expression of IL-10, lymphocyte activation gene 3 (Lag-3), PD-1 and PD-1 ligands [170]. Patients with ccRCC were also classified by immune- and inflammation-related gene signature using TCGA cohorts and determined three groups: (1) T cell-enriched (2) heterogeneously infiltrated and (3) non-infiltrated groups. The group with enriched T cells exhibited increased expression levels of granzyme B and INF-γ, PD-1 and cytotoxic T-lymphocyte-associated protein 4 (CTLA-4). This cluster corresponded to the worst survival. The T cell enriched group was further divided into two subgroups, called *TCa* and *TCb*. Gene expression of *TCa* was linked to metabolic and mitochondrial processes and better survival, while *TCb* was associated with cell cycle, extracellular matrix, cellular proliferation, and macrophage infiltration via stromal cells. Moreover, the infiltration of specific T cell subsets had prognostic significance, as Th17 cells and positive CD8^+^ T/T_reg_ ratio compared to Th2 cells and T_regs_ corresponded to an improved prognosis [166,167]. Based on the immune cell profile of TME, RCCs were classified into three groups: (1) immune-silent, (2) immune-regulated and (3) immune-activated RCCs. The immune-silent group of RCCs displayed T cells with low activation markers while the immune-activated group showed oligoclonal cytotoxic T cells (CD8^+^/PD-1^+^), and immunoglobulin and mucin-domain containing-3 protein positive T cells (Tim-3^+^). The hallmarks of the immune-regulated group included an inflammatory microenvironment with the infiltration of polyclonal exhausted CD8^+^/PD-1^+^/Tim-3^+^/Lag-3^+^ tumor-infiltrating leukocytes (TIL) lacking in cytotoxicity, linked to infiltration of dysfunctional DCs. In addition, this group was enriched in CD4^+^/ICOS^+^/Glucocorticoid-induced tumor necrosis factor receptor-related protein (GITR)^+^/Helios^+^ T cells displaying a T_reg_-cell-like phenotype expressing CD25^+^/CD127^-^/Foxp3^+^ [168]. Ultimately, immune-regulated tumors exhibited the highest risk of post-surgical progression, however, also present obvious targets for ICIs [171]. PD-1 expression was widespread among several T cell subsets, as well as CD38, a cyclic ADP-ribosyl hydrolase 1 associated with inflammation, autoimmunity and hematologic malignancies [172], proposed as a new marker of T cell exhaustion [163]. The expression of further co-stimulatory receptors or markers of T cell exhaustion, however, varied among the subsets, such as Tim-3, tumor necrosis factor receptor superfamily 9 (4-1BB), CTLA-4 and tumor necrosis factor receptor superfamily member 4 (OX-40). 

Tumor-associated macrophages (TAMs) are heterogeneous immune cell population in the TME. Clustering analysis of TAMs based on surface proteins showed different groups aligning to three branches. Apart from clusters corresponding to transitionary and circulating CD14^+^ and inflammatory CD16^+^ monocytes, these studies revealed a branch of mature TAMs only detectable in tumors of grade II or higher. The macrophages in these clusters exhibited diverse expression patterns, such as inflammatory marker CD38, as well as ones corresponding to a pro-tumor (CD163, CD204, CD206) and anti-tumor (CD169) phenotype. Furthermore, a CD68^+^/CD38^+^ macrophage population colocalized with exhausted CD8^+^/CD38^+^ T cells with elevated levels of PD-1 [163]. As the macrophage population also express PD-1 ligands such as programmed cell death 1 ligand 2 (PDCD1LG2) and PD-L1, it was proposed that these cells play a role in the establishment of an immunosuppressive TME in ccRCC [163]. Studies analyzing single-cell protein activity found that RCC-resident macrophages expressed a unique protein signature correlating with a higher risk of post-nephrectomy regression [173]. Profiling of TME genes in patients with advanced localized and metastatic RCC responding to ICI revealed a polarization towards an M1-like macrophage. Although M1 macrophages are responsible for the elimination of pathogens or tumor cells, M2 macrophages exhibit immune suppressive properties and a tendency towards tissue repair and tumor progression [174,175]. Remarkably, upregulation of IFN signaling and CD8^+^ T cell immune checkpoint genes, was paralleled by anti-inflammatory genes including V-set immunoregulatory receptor (VSIR), V-set and immunoglobulin domain containing 4 (VSIG4), PD-L2 and sialic acid-binding Ig-like lectin 10 (SIGLEC10). This result suggests a dual pro- and anti-inflammatory role of TAMs in RCC after ICI treatment, which probably leads to either immune suppression or resistance to ICIs [176]. Studies based on single-cell RNA sequencing of immune cell and T cell receptors along a pseudotime trajectory, respective of the tumor stage, showed early pro-inflammatory macrophages, while later in pseudotime or at advanced tumor stages, macrophages displayed an anti-inflammatory (M2-like) and CD8^+^ T cells an exhausted signature. A composite expression signature between terminally exhausted T cells and tumor macrophages was associated with worse OS across all tumor stages. Interestingly, the response to ICIs did not correlate to the composite signature [177]. Therefore, these studies propose an interplay between T cells and TAMs resulting in inflammation and defective immune response in RCC, leading to a reduced outcome.

In summary, several studies of the TME in RCC have revealed a crucial role in the interaction between immune cells, vasculature, and tumor cells in the development of tumors as well as in their responsiveness to and resistance to targeted therapy. Understanding the connections between key regulators of TME and interactions between various intratumoral cell types may increase therapeutic efficacy, particularly in RCC with more advanced stages.

## 9. Cytokine and Chemokine-Mediated Inflammatory Responses in RCC

### 9.1. IFNγ

Interferon-gamma (IFNγ) is an important cytokine that activates both the innate and adaptive immune systems. During the early phases of cancer, activated immune cells (NKs, CD8^+^, CD4^+^ T cells) secrete IFNγ, thereby modulating the activity of macrophages, DCs, Th1 CD4^+^helper T cells, and B cells to eliminate cancer cells. Therefore, IFNγ has anti-tumorigenic effects, which significantly suppress tumor growth. At the later step of tumor progression, IFNγ impairs secondary anti-tumor immune responses. However, long-term exposure to IFNγ can exert pro-tumorigenic effects inducing immunosuppression, angiogenesis and tumor cell proliferation [178]. IFNγ can upregulate the expression of programmed-death ligand 1 (PD-L1) on the surface of cancer cells, which binds its ligand (PD-1) on the activated CD8^+^ T cells. This cellular interaction leads to apoptosis of CD8^+^ T cells, thereby reducing anti-tumor CD8^+^T-cell cytotoxicity and creating an immunosuppressive TME. In addition, IFNγ also increases the expression of checkpoint inhibitors, such as indoleamine-2,3-dioxygenase 1 (IDO1) and CTLA-4, leading to enhanced tumor growth [179]. However, this was dependent on the RCC subtype, as PD-L1 in ccRCC showed a favorable effect on OS and DFS [180].

Other IFNγ-regulated gene is RBCK1, which showed markedly differential expression levels in cancer, and significantly correlated with tumor-infiltrating immune cells, tumor purity, and immune checkpoint molecules, such as PD-L1, CTLA-4, Lag-3, and T-cell immunoglobulin and ITIM domain (TIGIT) in pan-cancer samples [181]. High expression levels of RBCK1 displayed an immunosuppressive phenotype with decreased numbers of infiltrating CD4^+^ T cells, CD4^+^FOXP3^+^ Treg cells, M1 macrophages, and CD56^bight/dim^ NK cells, and was associated with a worse prognosis in RCC patients. Furthermore, RBCK1 promotes p53 degradation through ubiquitination, which further enhances RCC progression.

Interestingly, tumor-associated activation of NF-κB represents a protective mechanism against IFNγ-mediated pathways. In the absence of NF-κB, IFNγ can trigger receptor-interacting protein kinase 1 (RIPK1)-dependent programmed necrosis (also called necroptosis) in resistant RCC cells [182]. Interestingly, treatment of cells with IFNγ/CD70 conjugated antibody could induce RIPK1-dependent necroptosis in RCC cells in the presence of NF-κB inhibitor bortezomib [183]. Although this result opens a gate for a new therapeutic approach, future studies are needed to determine the preclinical potential of this treatment. 

FGF and FGFR1 are highly expressed in primary RCCs, and could enhance tumor cell proliferation, survival, and angiogenesis [184,185]. Activation of the FGFR signaling inhibits IFNγ-induced STAT1 phosphorylation and the expression of PD-L1, thus protecting cancer cells from the cytotoxic effects of CD8^+^ T cells [186], thereby supporting resistance to anti-cancer therapies [185]. In a mouse model of RCC, lenvatinib treatment inhibits both EGFR and FGFR signaling, and could restore the IFNγ-STAT1 signaling pathway and consequently prove the anti-cancer effect of the inhibitor [186,187].

In mammalian tissues, the essential amino acid tryptophan is metabolized by the kynurenine pathway. Kynurenine metabolites inhibit T cell proliferation and NK and T_reg_ cell activities [188]. IDO1 is involved in this metabolic pathway, converting tryptophan to N-formyl-kynurenine. Interestingly, IDO1 is highly expressed in different cancer cell types, therefore IDO1 inhibitors were proposed as a potential therapeutic approach for the treatment of cancer [189]. IFNα therapy in RCC could further induce IDO1 activity, showing elevated tryptophan metabolic products in the urine of RCC patients [190], which indicates impaired immune responses. The combined therapy of IFNα and IDO1 inhibitors resulted in a strong beneficial effect on RCCs, because IFNα could properly stimulate the immune system, while the immunosuppressive activities mediated by IFNα/IDO1 signaling were blocked [189]. 

IFN effectors such as IFNGR1/2 and STAT1/2 are associated with metastatic RCCs and predict poor OS [191,192]. IFNγ downstream effector IFIT5-XRN1 is found to degrade tumor suppressor miRNA (miR-363 and miR-128) resulting in a pro-tumorigenic axis, which upregulates EMT drivers, such as Slug and zinc finger E-box binding homeobox 1 (ZEB1) [191,193]. Altogether, these results suggest that IFN-regulated genes can potentially be used as biomarkers to predict the efficacy of ICIs. 

### 9.2. IL-2

Interleukin 2 (IL-2) stimulates the proliferation and cytolytic potential of NK and CD8^+^ T cells and promotes antibody release by B cells [194]. IL-2 binds and activates different receptor complexes, containing alpha (IL-2Rα, CD25), beta (IL-2Rβ, CD122), or common gamma chain receptors (γ_c_, CD132). The CD8^+^ T cell expansion is mediated through the heterodimeric IL-2Rβγ complex while the expansion of immunosuppressive CD4^+^ CD25^+^ T_reg_ cells is regulated by the IL-2Rαβγ complex. PEGylation has been used to conjugate polyethylene glycol (PEG) to IL-2. The PEGylated form of IL-2 can bind these receptor complexes and improves CD8+ T cell induction with minimal toxicity [194]. Although PEGylated IL-2 has been tested in clinical trials and the therapeutic effects were compared to nivolumab to cabozantinib or sunitinib, results are still pending (NCT03729245). 

GI101 is a molecular agent comprising the extracellular domain of CD80 together with a long-acting IL-2 variant that preferentially binds to the IL-2Rβ complex [195]. In a preclinical setting, GI101 strongly stimulates CD8^+^ T and NK cell proliferation without affecting T_reg_ cell population. GI101 elicits improved restoration of immune functions compared to a CD80-Fc without IL-2-associated toxicity [196]. A phase 1/2, open-label, dose-escalation and expansion study has been designed to evaluate GI101 as a single agent or in combination with pembrolizumab, lenvatinib or local radiotherapy in advanced and metastatic solid tumors (NCT04977453).

### 9.3. IL-12

Antigen-presenting cells produce interleukin 12 (IL-12) in response to infections, which promotes CD4+ T cell differentiation into Th1 cells. IL-12 has a positive synergistic proliferative effect on pre-activated NK and T cells and enhances independently and/or synergistically the cytolytic activity of both immune cell types by upregulating genes encoding cytotoxic cell granule-associated proteins [197,198]. Although preclinical studies proposed IL-12 as a potent immunotherapeutic agent in cancer therapy, disappointing clinical study was reported about the human recombinant IL-12 (rIL-12) treatment, and the negative result was explained by the inability of rIL-12 to reach the TME with effective doses [199]. The rIL-12 treatment also resulted in high levels of pro-inflammatory cytokine production, thereby inducing adverse side effects [200]. Therefore, novel approaches were developed to obtain biologically relevant rIL-12 treatment. Immunocytokine fusion protein of NHS-IL12 was designed, which consists of the tumor necrosis-targeting NHS76 antibody and fused to the human IL-12 heterodimer. This strategy was successful to deliver IL-12 to the necrotic region of the tumor with limited systemic exposure and associated toxicity [201]. Although NHS-IL12 was initially well tolerated by patients, a later study in combination with the anti-PD-L1 antibody avelumab was terminated (NCT02994953), due to the lack of observable efficacy. IL-12 fusion protein with humanized antibody BC1 was developed to target the ED-B splice variant of fibronectin, which is only expressed in the tumor-associated vasculature. Although the drug was well tolerated in the phase I evaluation of RCC, the cohort size was low (n=2) [202]; therefore, follow-up clinical trials are necessary for the final conclusion.

Tumor-targeted oncolytic viruses represent a long-promised revolution in cancer immunotherapy, because viruses can selectively target and eliminate tumor cells. The virus-infected cancer cells (or cancer lysates) release tumor antigens and intracellular proteins, which induce anti-tumor immune responses through IL-12-mediated pathways [203]. Genetically modified adenovirus with triple gene deletions is a safety tumor-sensitive virus vector (Ad-TD-LUC) for cancer cell targeting, and it was used to deliver a modified IL-12 (nsIL-12) to pancreatic cancer [203]. nsIL-12 lacks the N-terminal signal peptide, thereby preventing IL-12 secretion from the intact cell, but it can be released by necrotic cancer cells. Adenovirus-nsIL-12 treatment significantly improved outcomes, compared to mice treated with adenovirus-IL-12, which succumbed to IL-12-induced toxicity [203]. Further studies are necessary to evaluate this treatment in RCCs.

### 9.4. IL-6/JAK/STAT3 

Interleukin-6 (IL-6) is required for inflammatory reactions induced by TNF, IL-1, INF, and bacterial or viral infections [204]. IL-6 is produced by tumor cells and cells in the TME, such as fibroblasts, T and B cells, as well as myeloid, endothelial, and mesangial cells [205,206,207]. IL-6 binds IL-6 receptor (IL-6R), forming IL-6R/gp130 receptor complex which activates Janus-kinase (JAK). JAK subsequently phosphorylates tyrosine residues of gp130, thereby resulting in the activation of the gp130 Tyr^759^-derived Scr homology 2 domain-containing protein tyrosine phosphatase-2 (Scr-2)/extracellular-signal-regulated kinase (ERK)/mitogen-activated protein kinase (MAPK) pathway and the gp130 YXXQ-mediated JAK/ STAT pathway [205]. 

During cancer progression, JAK/STAT3 signaling enhances tumor proliferation, survival, invasion, immune evasion, and metastasis [208]. STAT3 was identified as the major mediator of IL-6-dependent cancer cell proliferation in RCCs [209]. In genomic and transcriptional profiling studies of RCCs, JAK/STAT3 gene signature was associated with a poor outcome and was correlated with an increased immunosuppressive T_reg_ cell score and served as an independent prognostic factor in RCCs [210]. 

In the kidney, different cell types can synthesize IL-6, which confers a pro-inflammatory phenotype to kidney diseases [211,212,213,214,215,216,217,218,219,220,221]. Serum levels of IL-6 correlated with the tumor stage of the RCC and was therefore considered a prediction factor of disease recurrence, progression, and OS and disease-specific (DS) survival [222,223,224,225,226,227,228]. High expression levels of IL-6 were detected in endothelial and vascular smooth muscle cells, indicating the vascular origin of IL-6 in patients with RCC. Furthermore, tumor-associated CD4^+^ T cells can also release IL-6 in ccRCC [226,229]. 

Hypoxia increases IL-6 production through the NADPH oxidase 4 (Nox4) signaling pathway in RCCs [23]. The HIF1-mediated VEGF expression is modulated by STAT3 signaling in RCCs [35]. High levels of IL-6 promote tumor invasiveness, and it is strongly associated with reduced response to sunitinib and pazopanib in patients with metastatic RCC [230]. This tumor resistance was explained by the ability of IL-6 to induce the phosphorylation of tumor AKT, mTOR and NF-κB signaling [231]. Activation of p21-activated kinase 1 (PAK1) influences IL-6/NF-κB activation, leading to a stem-like phenotype and consequent sunitinib resistance in RCCs [232]. An alternative mechanism also promotes the proliferation of RCCs through the downregulation of hepatocyte cell adhesion molecule (hepaCAM). Activation of IL-6/STAT3 signaling results in epigenetic changes (hypermethylation) in hepaCAM promoter and consequent inhibition of gene expression [233].

In VHL-deficient RCCs, IL-6 production is increased, which propagates macrophage migration to the tumor site and polarization toward an M2-like phenotype [55]. IL-6-activated macrophages release TGFβ; therefore, renal tubular epithelium develops an adenoma-like phenotype. IL-6 also activates human CD14+ peripheral blood nuclear cells through the STAT3 pathway producing CCL18 and TGFβ, which leads to the induction of the EMT with tumor-promoting effects. Therefore, tumorigenic EMT seems to be promoted by a reciprocal immunosuppressive interplay between tumor cells and macrophages, activated by IL-6/JAK/STAT3 signaling during the early phases of tumor progression [55]. In ccRCC, it has been proposed that cytotoxic T cell activation and consequent response to immunotherapy are probably impaired by enhanced IL-6/JAK/STAT3, IFNα, IFNγ signaling routes and RIPK2-mediated signaling [234,235]. Altogether, these results suggest that direct blockade of IL-6 or its downstream effectors could be a potential strategy to cure RCCs.

Siltuximab is a chimeric mouse–human monoclonal antibody that binds and blocks IL-6 functions. Siltuximab has been tested in a clinical trial (NCT00265135) with beneficial effects, stabilizing progressive metastasis in half of the patients with RCCs [236]. IL-6R and JAK inhibitors have been used in the treatment of inflammatory diseases, including myeloproliferative neoplasms and the management of adverse effects of CAR-T cells [207]. Tocilizumab is a recombinant humanized monoclonal antibody targeting IL-6R, approved for the treatment of arthritis [237,238,239,240] and giant cell arteritis [241], CAR-T cell-induced cytokine-release syndrome [242], systemic sclerosis-associated interstitial lung disease [218,243,244], and severe acute respiratory syndrome coronavirus 2 (SARS-Cov-2 infection) [245,246]. Tocilizumab has been proposed to support the anti-tumor effect of IFNα treatment in RCCs by inhibiting the IL-6-mediated expression of the suppressor of cytokine signaling 3 (SOCS3) [247,248]. In a xenograft mouse model, tocilizumab therapy was combined with TKI and beneficial inhibitory effects were observed in tumor growth and vascularization, compared to TKI monotherapy, implicating a positive clinical evaluation [231]. 

### 9.5. IL-1β

Interleukin 1 beta (IL-1β) is an inflammatory agonist that has many immunomodulatory effects in the kidney [249]. Both tumor and stromal cells can release IL-1β, generating an immunosuppressive TME enriched in TAMs, myeloid-derived suppressor cells (MDSCs), and T_reg_ cells [250,251,252]. IL-1β in the TME reduced tumor growth, invasiveness and angiogenesis, and facilitated checkpoint inhibition [250,251,252,253]. IL-1β-mediated expansion of MDSCs in ccRCC tumor is also associated with elevated intratumoral levels of angiogenic IL-8, CXCL5 and chemotactic C-C motif chemokine 3 (also known as MIP-1α) [254]. A recent study showed increased efficacy of TKI, cabozantinib, or anti-PD-1 checkpoint-inhibition, when combined with an anti-IL-1β mAb in a heterotopic mouse model of RCC [255]. The number of intratumoral immunosuppressive MDSCs was strongly TAMs differentiated toward an anti-cancer (M1-like) phenotype [255]. 

Additionally, pathogen- and damage- associated molecular patterns (PAMPs, DAMPs), and NLR family pyrin domain containing 3 (NRLP3)-inflammasome activation in TME may cause the production of IL-1β. Although IL-1β levels in RCC tumors are frequently increased, it is still an open question whether this inflammasome activation may affect the growth and metastasis of RCC [249]. Drugs used in RCC patients may further drive inflammasome activation [249].

### 9.6. TNFα

Tumor necrosis factor α (TNFα) is a key mediator of cancer-related inflammation in RCCs, promoting a pro-tumoral milieu by inducing the production of reactive oxygen intermediates, cyclooxygenases, matrix metalloproteinases (MMPs), and cytokines [256,257]. Serum and intratumoral levels of TNFα are strongly elevated in RCCs, compared to other cancer types, and correlated with tumor size [258,259]. Gene expressions of chemokine receptors (CXCR2 or CXCR3) are induced by TNFα and associated with a worse outcome in a high-risk cohort of patients with ccRCC. TNFα also enhances PI3K/AKT signaling and GSK-3β signaling-dependent tumor necrosis factor α-induced protein 8 (TNFAIP8) and MMP-9 expression. The elevated mRNA expression of the EMT-associated transcriptional factors Slug and ZEB1 and increased expression of vimentin, a mesenchymal marker over epithelial marker E-cadherin, resulting in enhanced migratory and invasive potential [260,261,262,263,264,265]. The co-expression of TNFα with MMP-9 or cancer stem cell marker CD44 in metastatic RCC tumors increases the resistance to sunitinib treatment [259,266]. TNFα also has an indirect effect on adaptive immune response by inhibiting T cell activity in the TME. Tumor-derived gangliosides inhibit anti-tumor efficacy via altering TNF-induced NF-κB activation in T cells and inducing T cell apoptosis [267]. TNFα has anti-proliferative effects in RCC when combined with Th2 cytokines IL-4 or IFNγ, highlighting its complex role in the regulation of immune response [268,269,270]. In the early 2000s, a series of phase II clinical studies investigated infliximab, a chimeric anti-TNFα monoclonal antibody. The trials first demonstrated promising results in low and high-dose treatment protocols of chemokine-resistant patients but eventually failed in combination therapy with first-line TKI treatment, producing side effects without observable improvement [271,272]. Studies on TNFα in RCC therapy have stepped back in favor of TKIs, mTOR inhibitors, and other approaches.

### 9.7. TGFβ

Transforming growth factor beta (TGFβ) signaling is involved in a variety of physiological and pathophysiological processes, including proliferation, migration, differentiation, apoptosis, embryonic development, tissue repair, angiogenesis and inflammation [273]. In the canonical Smad-dependent pathway, TGFβ ligands bind to serine/threonine kinase receptor TGFβ receptor type II (TβRII), leading to the recruitment and phosphorylation of TGFβ receptor type I (TβRI, ALK5, ALK5-Full Length/ALK5-FL). Downstream effectors, Smad-family member 2 and 3 (Smad2/3) are phosphorylated, resulting in a complex formation including Smad4, and activation of target genes [274,275,276]. The non-canonical (pathway involves translocation of the TβRI-intracellular domain (TβRI-ICD, ALK5-ICD) into the nucleus, independent of Smad [277]. TGFβ is considered as an anti-inflammatory cytokine in the context of renal inflammation [278]. However, TGFβ regulates renal fibrotic diseases as well, inducing the production and abnormal accumulation of extracellular matrix components [279,280,281,282,283,284,285,286]. TGFβ signaling is quite complex in kidney diseases since TβRII deficiency in kidney fibroblasts and tubular epithelial cells impairs TGFβ/Smad3-mediated kidney fibrosis, but simultaneously enhances NF-κB-mediated renal inflammation via upregulation of IL-1β and TNFα-mediated pathways [287]. 

Elevated serum levels of TGFβ are associated with metastatic disease [288]. Tissue expression of TGFβ correlates to tumor stage, histological type and nuclear grade [289]. Increased activity of TGFβ-mediated pathways, as a prognostic signature, was detected in patients with ccRCC [275]. TGFβ/Smad2/3 signaling leads to improved invasiveness of RCCs [290]. TβRII and TβRIII deficiency have been associated with primary tumor formation and metastases of RCCs, respectively [291,292]. A ubiquitin-dependent degradation of TβRII was observed in advanced RCCs which is regulated by the E3 ligase Smad-ubiquitination regulatory factor 2 (Smurf2), thereby dysregulating TGFβ signaling pathways [293]. Cytoplasmic TβRIII domain-induced apoptosis functions through a TβR/Smad-independent manner involving the p38-MAPK pathway [294]. Treatment of RCC cell lines with recombinant TGFβ (rTGFβ) leads to the downregulation of cadherin 1 and claudin 1 (epithelial markers), and the upregulation of vimentin, ZEB1 and zinc-finger transcription factor Snai1 (mesenchymal markers) in a TGFβ/Smad-dependent manner, highlighting the contribution to EMT formation. rTGFβ treatment could downregulate TβRII and upregulate TβRI expressions in ccRCC, which is probably consistent with the link between enhanced tumor growth and TβRII deficiency [295].

Analyzing ccRCC cohorts, an increased tumor immune infiltration was observed and correlated with activation of TGFβ pathway [296]. Suppression of TGFβ-induced paired box gene 2 (PAX2) or SMAD proteins binding to the PAX2 promoter has been suggested to be involved in EMT of RCCs [297]. Integrin signaling potentiated TGFβ1-induced E-cadherin depletion in vitro, mediated by the EMT-associated transcription factor Snai1 [298]. Forkhead activin signal transducer 1 (FAST1, FOXH1) is upregulated in RCC and mediates activation of the TGFβ/Smad signaling pathway, leading to increased cell viability, proliferation, migration, invasion, EMT, and reduced apoptosis as well as increased tumor growth [299]. Neutralizing TGFβ in a heterotopic cancer model decreased the size of tumors and inhibited vascularization [300]. TGFβ-activated kinase 1 (TAK1)-inhibitors could also inhibit cancer cell survival by impairing NF-κB activation and inducing apoptosis [301]. High mRNA levels of IL-10 and TGFβ were detected in the peripheral blood of metastatic RCC patients compared to the healthy control group. Although various T cell subsets could release TGFβ, mainly monocytes were responsible for TGFβ production in the peripheral blood, and this was correlated with favorable prognosis for patients with metastatic RCCs [302]. Exosomes released from primary tumor cells also contain TGFβ, thereby attracting tumor-infiltrating NKs by regulating the TGFβ/Smad pathway [303]. In a preclinical study, tumor-reactive CD8+ T cells were isolated from RCC patients, activated with autologous immature DCs and tumor lysate, and infected with a TβRIIDN retrovirus to inhibit TGFβ signaling. RCC patient-derived peripheral blood mononuclear cells were injected into immunodeficient RCC-xenografted mice, followed by the transfer of autologous patient-derived TGFβ-resistant CD8+ T cells. The autologous TGFβ-insensitive CD8+ T cells displayed cytotoxic activity and decreased tumor burden and lung metastasis, and increased survival [304].

Targeting of TGFβ was extensively investigated in various kidney diseases, such as focal segmental glomerulosclerosis and diabetic nephropathy [285,305,306]. Several studies suggested an immune suppressive role of TGFβ; therefore, many agents blocking downstream effectors of TGFβ have been investigated in both preclinical and clinical studies. Low-dose chemotherapeutic drugs initially inhibited TβRI expression on tumor cells but promoted IFNγ secretion and T-cell infiltration into tumors. At later stages, this effect was reversed, as the TβRI and Smad expression were increased in cancer and T cells [307]. Salinomycin is a monocarboxylic polyether antibiotic against Gram-positive bacteria. Moreover, salinomycin also kills cancer stem cells and potentially inhibits cancer growth and metastasis through DNA damage and inhibition of TGFβ-mediated EMT [308]. Salinomycin also inhibits the proliferation and viability of RCC cell lines. TβRI inhibitor reduces tumor cell invasion by attenuating the TGFβ/Smad3 pathway [309].

Two types of estrogen receptors (ERα, ERβ) regulate cancer development and interestingly, RCC cell lines only expressed the ERβ isoform [310]. Increased ERβ expression was detected during RCC development, and this was associated with worse survival for RCC patients [310]. In a mouse model of RCC, ERβ supported cancer cell proliferation, migration and invasion, and could affect the expression of TGFβ/Smad3 signaling to control cancer cell invasion [311]. In line with these results, anti-estrogen (ICI 182,780) and selective ERβ antagonist (PHTPP) could effectively inhibit tumor growth and invasion in an orthotopic mouse model of RCC [310].

Pirfenidone is a pyridone compound with anti-fibrotic effects due to the downregulation of TGFβ, PDGF and type I collagen synthesis [312,313,314]. Therefore, pirfenidone has been proposed to treat patients with kidney and pulmonary fibrosis. Pirfenidone inhibits TGFβ-mediated EMT, migration, proliferation, and invasion of RCC cells through decreasing Smad2/3 phosphorylation. In a subcutaneous RCC model, pirfenidone could inhibit tumor growth and recruitment of MDSCs [315].

Fresolimumab is a pan-TGF-targeting neutralizing monoclonal antibody, which was subjected to clinical trials, including advanced RCCs and malignant melanoma (NCT00923169) [316]. Fresolimumab displayed an acceptable safety profile and anti-tumor activity [317]. However, many patients did not exhibit increased OS, due to the reduced expression of activating surface proteins in NK cells [318]. Another anti-TGFβ antibody NIS739 was also studied in a phase I/Ib trial as a single agent or in combination with spartalizumab, an anti-PD-1 antibody (PDR001) in patients with RCC and advanced malignancies. Results showed synergic effects in attenuating immunosuppressive responses to α-PD-1 (NCT02947165) [318,319]. Altogether, these results suggest that a TGFβ signalosome could be an attractive therapeutic target in the advanced stages of RCC.

### 9.8. Chemokine Receptors

Activation of chemokine receptors (CXCR1 and CXCR2) has pro-angiogenic and pro-inflammatory effects, inducing several signaling pathways, such as the protein kinase C, phospholipase C, PI3K/AKT/mTOR, RAS/RAF/MEK/ERK and NF-kB pathways, promoting tumor cell survival, proliferation, and dissemination [320]. The growth-related oncogene (GRO) subgroup CXCL1/GRO-a, CXCL2/GRO-b, CXCL3/ GRO-c, preferentially bind to CXCR2, while CXCL8 binds CXCR1. These interactions increase neutrophil recruitment to the tumor site and consequent VEGFA production and angiogenesis [321]. Blockade of CXCR1/2-ELR^+^CXCL interactions in malignancies appears to be an attractive therapeutic approach. Reparixin is a CXCR1/2 inhibitor and showed promising results in pilot studies of early and metastatic breast cancer and was evaluated in combination with paclitaxel [322] (NCT02370238).

Analysis of pan-cancer databanks showed that RCCs can expose the highest amounts of C-X-C motif chemokine receptors CXCR1 and CXCR2 [323]. High levels of chemokine expression correlated to the significantly worse OS and progression-free survival (PFS) in RCC patients [323]. Chemokine receptor expression in RCCs has a significant impact on the formation of a pro-inflammatory landscape in the TME. Dufies et al. investigated the effects of various CXCR1/2 blocking agents in ectopic models of RCC and identified compound C29 to inhibit primary tumor cell growth and ELR+CXCL-mediated proliferation and migration of endothelial cells [323]. The impact of C29 was equal in RCC cells that had not been exposed to TKIs and those that had, indicating a common method to prevent angiogenic escape. Further characterization of C29 effects in a pre-clinical study will unveil its potential in the therapy of RCC [323].

CXCR4 binds macrophage migration inhibitory factor (MIF), a factor implicated in the control of innate immunity, as well as CXCL12, a molecule well-known for its function in immunological surveillance, inflammatory response, tissue homeostasis, and tumor development and metastasis [324,325]. CXCR4 expression levels especially increased during the invasive phases of cancer involving EMT formation. In patients with RCC, high CXCR4 expression correlates with worse OS and PFS [326]. High-grade RCC samples showed a signature of CXCR4, CXCR7, VIM, CDH2 and ZEB1, while metastatic samples had even higher expression levels for VIM, CXCR4 and FN1 [327]. VHL deficiency or aberrant β-Catenin (*CTNNB1*) expression induced CXCR4 expression in RCCs. CTNNB1 is a component of the Wnt signaling pathway, which regulates tumor growth and metastasis through the expression of ICAM-1, VCAM-1, CXCR4, and CCL18 [328].

In mouse models, interfering with the CXCR4–CXCL12 axis promoted CD8^+^ T cell infiltration and reduced CXCL12-mediated migration of lymphocytes and MDSCs to the TME, as well as inhibited tumor revascularization following anti-angiogenic therapy [320]. Multiple studies currently evaluate X4P-001 (mavorixafor), a CXCR4 inhibitor, in combination with standard therapy options. The combined therapy of X4P-001 with VEGF inhibitor axitinib is well tolerated in patients with advanced RCC and demonstrates encouraging median PFS [329] (NCT02667886). A phase 1b trial of mavorixafor and ICI nivolumab in advanced RCC patients with no prior response to nivolumab monotherapy showed promising tolerability and anti-tumor activity [330].

## 10. Conclusions

RCC can develop from different genetic insults, including as a consequence of kidney injury. Many hallmarks of RCC are mediated by inflammation, including tumor cell proliferation, migration, angiogenesis, and tumor spread. Inflammation can be also due to extrinsic factors, such as obesity, infection, and exposure to pollutants, bacteria, viruses, and tobacco, which can increase the susceptibility to developing RCC. Although eradication of inflammation-causing factors as earlier as possible is an optimal approach, this requires robust diagnostic markers. Inflammation is significantly reflected by quantitative and qualitative alterations in RCC’s immune cell landscape, such as immune cell infiltration and immunosuppression. Testing systemic inflammation markers in blood and urine samples of RCC patients, including circulating leukocytes, immune cells and inflammatory cytokines, provide important prognostic data. However, predictive accuracy depends on the patient profile, which has to be taken into the account, i.e., patients who already received immunotherapy. In this case, pre- and post-therapeutic assessment of inflammation-related markers can better predict response and survival in patients undergoing treatment. Single-cell RNA-sequencing approaches in clinics offer many benefits; it can determine the source, origin and type of inflammation-inducing cells and identify interconnected resistance pathways, thereby proposing powerful risk-stratification models. Due to the differences between mouse and human RCC oncogenesis, many stochastic mouse RCC models do not mimic all the stages of tumor development and fail to form metastases. Although patient-derived xenograft models retain many characteristics of human patients and serve as valuable tools to dissect specific mechanisms, they lack an immune system. Therefore, additional experimental studies in the field are required to translate the findings from animal models to human RCC. Understanding the intricate inflammatory pathways controlling tumor cell or TME plasticity may help to identify new therapeutic targets in RCC in order to better design personalized medicine approaches.
